# Letter to the Editor

**DOI:** 10.1080/20018525.2016.1270077

**Published:** 2017-01-05

**Authors:** Jean Bousquet

**Affiliations:** ^a^ Jean Bousquet University Hospital, MACVIA-France, Montpellier, France

Dear Professor Bjermer:

I have read with great interest the review article ‘Severe asthma: anti-IgE or anti-IL-5?’ by Papathanassiou et al. [1]. I found it very relevant because the multiplicity of new biologics in severe asthma will require a better patient stratification using clinical tools [2] to estimate the best treatment choice for individual patients. One of the clinical end points which should be considered is oral corticosteroid (OCS) usage.

The paper is comprehensive and also provides an overview of the oral steroid-sparing effect of mepolizumab. It is worth mentioning that the OCS reduction has also been observed in patients receiving omalizumab. In a randomized clinical trial (EXALT study),[3] the change from baseline in mean maintenance OCS dose at Week 32 was significantly greater in the omalizumab group than in the optimized asthma therapy (OAT) group (−45% vs. + 18.3%, *p* = 0.002). In the omalizumab group, 37 patients (62.7%) reduced/stopped OCS use at Week 32, compared with seven patients (30.4%) receiving OAT (*p* = 0.013). These results demonstrate the OCS-sparing effect of omalizumab in patients enrolled in the EXALT study.[4]

In addition, OCS reduction in patients treated with omalizumab has been confirmed in multiple studies in the real-life setting, such as the multinational eXpeRience study [5] and the studies conducted in the UK (APEX I and II [6,7]).

Therefore, it should be noted in this article that although an oral steroid-sparing effect has been observed in clinical trials of mepolizumab, this has also been demonstrated in both clinical trials and real-life studies of omalizumab, particularly as the review attempts to provide an overview of which agent is more suitable in the management of severe allergic asthma.
